# Upcycling Wool Waste into Keratin Gel-Based Nanofibers Using Deep Eutectic Solvents

**DOI:** 10.3390/gels9080661

**Published:** 2023-08-17

**Authors:** Cláudia Mouro, Rodrigo Martins, Ana P. Gomes, Isabel C. Gouveia

**Affiliations:** FibEnTech Research Unit, Faculty of Engineering, University of Beira Interior, 6200-001 Covilhã, Portugal

**Keywords:** wool waste, wool keratin, deep eutectic solvent, electrospinning, gel-based nanofibers, waste valorization

## Abstract

Millions of tons of wool waste are produced yearly by textile industries, which may become a serious environmental hazard in the near future. Given this concern, it is crucial to explore strategies to reduce the amount of wool waste generated worldwide and adopt more sustainable practices for dissolving and regenerating wool keratin (WK) from textile waste. Most traditional methods involve the use of expensive, toxic, harmful, and poorly biodegradable compounds. To overcome these limitations and facilitate the reuse of wool waste through a cascade valorization strategy, researchers have started testing the use of deep eutectic solvents (DES) as a more sustainable and eco-friendly alternative for WK dissolution and regeneration. In this study, the potential of two different DES mixtures, Choline chloride (ChCl): Urea and L-Cysteine (L-Cys): Lactic acid (LA), was explored for dissolving wool waste. Subsequently, the gels obtained based on DES-WK were blended with polyvinyl alcohol (PVA) in different ratios to produce nanofibers using the electrospinning technique. The PVA/L-Cys: LA DES-WK proved to be the most effective DES mixture for fabricating WK gel-based nanofibers. Furthermore, their antioxidant and antimicrobial abilities were evaluated, thus confirming their bioactivity. The results obtained revealed that this approach to valorizing textile waste offers a unique avenue for the development of sustainable functional materials with potential applications in various biomedical and industrial fields.

## 1. Introduction

Keratin is one of the most abundant and underexploited fibrous proteins found in the epidermis of vertebrates, as well as in some epidermal appendages such as nails, hair, fur, hooves, feathers, wool, and horns [1,2]. Additionally, keratin is present in numerous wastes produced by the textile and poultry industries, as well as slaughterhouses, that are considered environmental pollutants and which pose a serious threat to human health and the natural ecosystem [1,3,4]. In this context, it is crucial to convert the keratin present in these wastes into high-value-added products such as biomaterials, composite materials, films, reinforcements, fertilizers, absorbents, and cosmetics [2,3,4]. Moreover, these bio-based products are viewed as more economically sustainable and environmentally friendly due to their renewable nature, biocompatibility, and biodegradability. As a result, researchers are focused on reusing and dissolving these wastes [2,3].

Recycling the millions of tons of wool waste produced annually by the textile industries has become a significant research challenge [3,4]. Wool keratin (WK) is insoluble and resistant to the most common solvents due to the presence of strong intra- and intermolecular disulfide bonds, hydrogen bonds, and van der Waals forces, making its dissolution and regeneration difficult [1,2,3,5,6]. Therefore, various approaches capable of cleaving the disulfide and hydrogen bonds, such as oxidation, reduction, acid–alkali, sulfitolysis methods, enzymatic hydrolysis, and the use of ionic liquids, have been explored to extract WK from wool wastes [1,2,3,6]. However, many of these methods have issues related to time consumption, high temperatures, and rigorous reaction conditions, and some involve expensive, toxic, harmful, and poorly biodegradable compounds [2,3]. Consequently, in recent years, green dissolution methods have been proposed. Among them, the use of deep eutectic solvents (DESs) is gaining attention for the dissolution and regeneration of WK from wool waste due to their good biocompatibility, biodegradability, non-toxicity, easy availability, and low price [3,4]. Deep eutectic solvents (DESs) are mixtures of two or more compounds that act as either hydrogen bond acceptors (HBA) or hydrogen bond donors (HBD). These solvents have significantly lower melting points than their individual components, enabling reactions to occur at lower temperatures and establishing hydrogen and van der Waals bonds with the wool structure [2,3,4,5]. Various types of DES have been investigated for transforming wool waste and regenerating WK.

For example, Moore et al. utilized Choline chloride (ChCl): Urea DES at a molar ratio of 2:1 to obtain DES-WK. The dissolution of WK was carried out at 170 °C for 30 min with stirring [7]. Similarly, Jiang et al. employed ChCl: Urea DES in a 1:2 molar ratio to dissolve wool fibers at 130 °C for 5 h and regenerate WK [3]. In another study, Wang et al. tested ChCl: Oxalic acid (OA) DES at a molar ratio of 1:2 for WK dissolution. The results showed that DES-WK exhibited higher solubility when a 5.0% weight ratio of wool to DES was used at 110–125 °C for 2 h [4]. Additionally, Okoro et al. recently used a mixture of 1.6 g L-Cysteine (L-Cys) and 20 mL lactic acid (LA) to solubilize wool waste. The effectiveness of L-Cys: LA DES in WK recovery from wool waste was confirmed without affecting its structure [2]. However, these studies focused solely on the dissolution and regeneration of WK using DES, emphasizing the need to develop strategies for reusing this sustainable biopolymer to achieve high-value materials.

In this study, WK was initially dissolved in two different DES mixtures, specifically, L-Cys: LA and ChCl: Urea DES mixtures. Subsequently, the resulting gels based on DES-WK were electrospun into nanofibers. Electrospinning is considered one of the most versatile, simple, rapid, flexible, and cost-effective techniques for producing functional micro/nanofiber materials for a wide range of applications [8,9,10]. However, the preparation of electrospinning solutions often involves the use of organic solvents that are harmful to human health and the environment, and generally display a highly volatile behavior, which impairs the adjust the evaporation rate of the solution and results in the drying of the jet at the needle tip during their flight towards the collector, before the deposition in collector as solid nanofibers [11,12,13]. Green solvents like DESs offer a potential alternative for electrospinning to overcome the drawbacks associated with traditional solvents, namely due to their non-toxic and non-volatile nature. Additionally, biopolymers such as WK are often challenging to electrospun on their own, and require blending with other polymers [14,15,16,17,18]. Among these polymers, Polyvinyl Alcohol (PVA) has been extensively investigated for producing nanofibrous materials due to its biocompatibility, biodegradability, non-toxicity, enhanced fiber-forming ability, and excellent solubility in benign solvents such as water [19,20]. Therefore, to the best of our knowledge, this study represents the first successful blending of PVA with different weight ratios of gels based on DES-WK for electrospinning into nanofibrous membranes, aiming to valorize wool waste.

## 2. Results and Discussion

### 2.1. Dissolution of WK into DES Mixtures

The wool waste was placed in contact with two different DES mixtures, i.e., ChCl:Urea molar ratio 1:2 and 1.6 g L-Cys in 20 mL of LA, and dissolved at 130 °C for 3 h, respectively, in order to evaluate the influence of the composition of the DES system on the dissolution of the WK. In this regard, it can be seen in Table 1 that 1.6 g L-Cys in 20 mL of LA displayed a higher WK dissolution efficiency, reaching a solubility of 68.83 ± 5.10% at 130 °C, whereas for ChCl:Urea in a molar ratio of 1:2, a solubility of 42.88 ± 0.83% was reached at 130 °C.

Previous studies have reported that both DES mixtures are able to dissolve wool and regenerate keratin from wool waste. In particular, Okoro et al. reported with respect to the L-Cys:LA DES that the use of the LA affected the bonds in the wool, forming hydrogen bonds with peptides and other wool functional groups [2]. These new hydrogen bonds allowed the L-Cys to easily penetrate and cleave the disulphide bonds, consequently promoting the formation of thiolate anions, which can promote further cleavage of the disulphide bonds in keratin chains, thus leading to higher wool solubilization. In turn, Jiang et al. revealed the potential to dissolve and regenerate WK using the ChCl:Urea DES by cleavage of disulfide bonds among amino acid side chains [3]. In addition, ChCl, a rich hydrogen bond acceptor, has been used to break intermolecular or intramolecular hydrogen bonds. Therefore, both DES mixtures have been demonstrated to be able to successfully dissolve WK, although the solubility of the wool waste can be significantly influenced by several factors, like the molar ratio of hydrogen bond acceptor and donor in DES system, the dissolution time, and the temperature range.

### 2.2. Determination of the Antibacterial Activity of the DES Mixtures and the Gels Based on DES-WK

**Figure 1:**
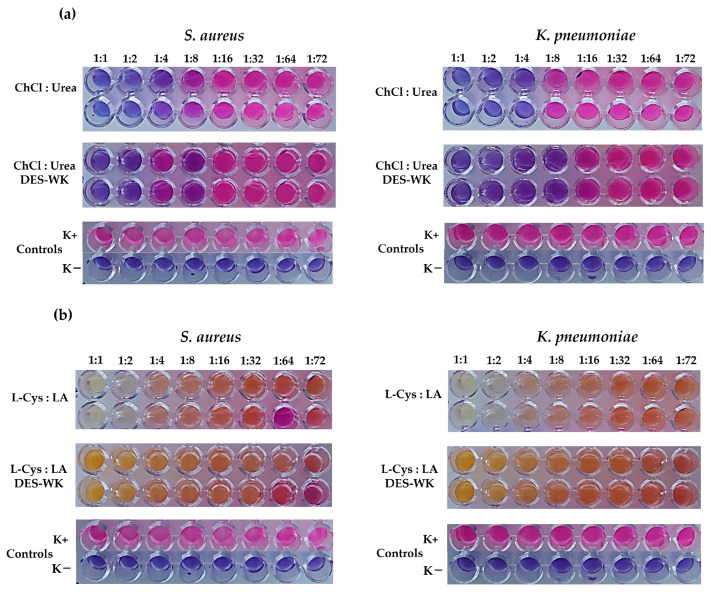
Determination of the minimum inhibitory concentration (MIC) of the ChCl:Urea DES and ChCl:Urea DES-WK (**a**); L-Cys:LA DES and L-Cys:LA DES-WK (**b**) against *Staphylococcus aureus* (*S. aureus*) and *Klebsiella pneumoniae* (*K. pneumoniae*), determined by resazurin-based 96well plate microdilution assay.

In our study, the lowest concentration of DES mixtures for preventing bacterial growth was defined as the minimal inhibitory concentration (MIC). The ChCl:Urea DES presented MIC values of 250 µL/mL for *S. aureus* and *K. pneumoniae*, corresponding to a 1:4 dilution; Figure 1a. In addition, it was found that the gel based on ChCl:Urea DES-WK exhibited a slightly higher antimicrobial activity, displaying MIC values of 125 µL/mL against *S. aureus* and *K. pneumoniae*, corresponding to a 1:8 dilution; Figure 1a. In turn, conclusive results were not obtained for the L-Cys:LA DES, since the blue-purple resazurin color changed to a colorless and/or orange/yellowish hue (Figure 1b), due to the resazurin chemical structural changes at different pH levels [21]. Ezati et al. reported that resorufin forms the caproyl ester group in acidic conditions, a colorless and non-fluorescent hexanoyl resorufin [21]. Moreover, Labadie et al. described that resorufin has an intense red fluorescence at neutral pH, while a yellow fluorescence is observed at pH below 6.6 [22]. Therefore, the resazurin reduction-based technique for detecting bacterial growth exhibited technical limitations related to the discoloration/color change in resorufin under acidic conditions due to the presence of LA in the DES mixture. However, both DES and the gels based on DES-WK dissolved exhibit recognized antimicrobial properties, as can be seen below in Section 2.4.5. Additionally, researchers have previously highlighted that DESs generally exhibit a higher antimicrobial activity than their individual components [23]. In addition, ChCl-based DESs have demonstrated broad-spectrum antibacterial properties, while L-Cys and LA are known to have antimicrobial activity [24,25,26].

However, DESs, as a new generation of green solvents, have attracted research attention due to their unique characteristics, which include lower toxicity, as well as biodegradability, environmental safety, and intrinsic bioactive properties [27,28].

### 2.3. Evaluation of the pH and the Electrospinning Solution Properties

#### 2.3.1. pH

**Figure 2:**
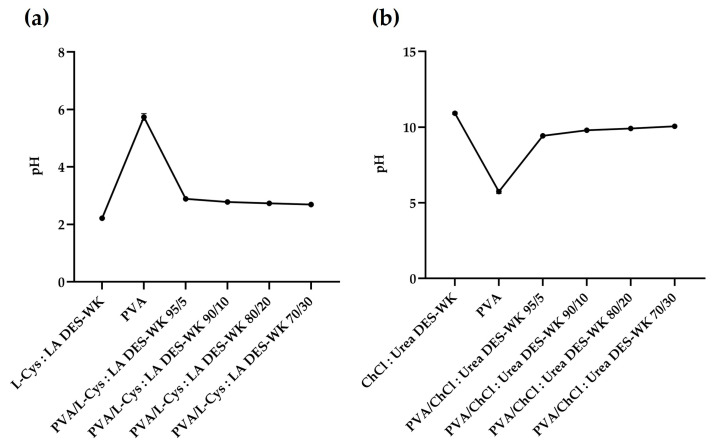
pH measurements of the PVA/L-Cys:LA DES-WK blend gel solutions (**a**) and PVA/ChCl:Urea DES-WK blend gel solutions (**b**), as well as the raw PVA and the gels based on DES-WK mixtures.

The properties of the electrospinning solutions, such as the electrical conductivity and the viscosity, can be influenced by the pH value [29]. In this regard, the pH of the PVA solution, the DES mixtures, and the PVA/DES-WK weight ratios of 95/5, 90/10, 80/20, and 70/30 were measured and recorded (Figure 2). The L-Cys:LA DES exhibited a pH value of 2.22 ± 0.17, while the PVA solution displayed a pH value of 5.74 ± 0.12, which is in agreement with the pH values reported in the literature for this polymer [30]. However, when the gel based on L-Cys:LA DES-WK was blended with the PVA in different ratios there was a slight increase in pH values compared to the L-Cys:LA DES mixture, Figure 2a.

In turn, the ChCl:Urea DES revealed a high pH of 10.92 ± 0.08, which resulted in an increase in the pH values of the PVA/ChCl:Urea DES-WK blend gel solutions relative to the PVA, Figure 2b.

Therefore, the effect of the pH on the electrical conductivity and viscosity of the electrospinning solutions, and consequently, on the production and the morphology of the electrospun nanofibers, is investigated in the following sections of this scientific paper.

#### 2.3.2. Electrical Conductivity

In the electrospinning process, a charged jet is ejected when the electrostatic repulsion forces, associated with an increase in the high voltage applied, overcome the surface tension of the electrospinning solution. At this moment, a liquid droplet is elongated into a conical shape forming a Taylor cone-jet, which is mainly controlled by the Coulombic force between the charges and the electric field [31,32,33]. Thus, these forces arise due to the surface charge on the jet and change according to the conductivity of the solution. As a result, the jet carries more charge as the electrical conductivity of the solution rises, and consequently, it is subjected to a greater elongation, resulting in uniform fibers with smaller diameters [31,32,33].

**Figure 3:**
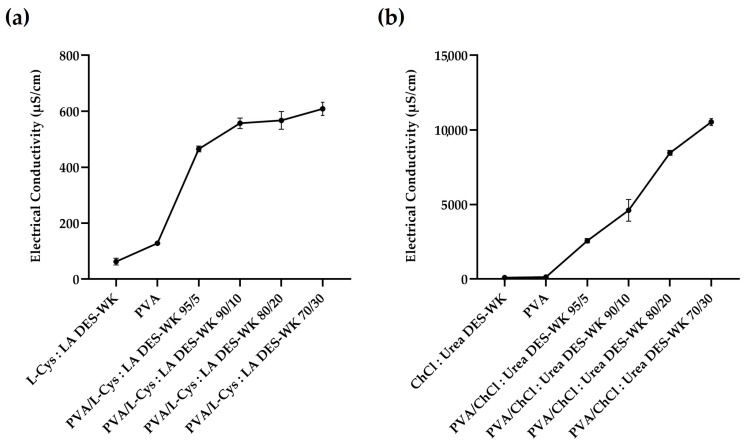
Electrical conductivity measurements of the PVA/L-Cys:LA DES-WK blend gel solutions (**a**) and PVA/ChCl:Urea DES-WK blend gel solutions (**b**), as well as the raw PVA and the gels based on DES-WK mixtures.

In this regard, the conductivity of the solutions used in the electrospinning, namely the gel-based DES-WK mixtures, the PVA solution, and the gel-based PVA/DES-WK blends with weight ratios of 95/5, 90/10, 80/20, and 70/30 were measured, and the results are recorded in Figure 3.

The L-Cys:LA DES-WK presented a low conductivity (62.58 ± 11.97 µS/cm) when compared to the PVA solution (128.43 ± 2.34 µS/cm), which is widely recognized by its ability to produce uniform nanofibers. Additionally, the PVA/L-Cys:LA DES-WK gel blends revealed higher conductivity values in comparison with both PVA and L-Cys:LA DES-WK, Figure 3a. Similarly, the gel based on ChCl:Urea DES-WK exhibited a lower conductivity (90.71 ± 20.48 µS/cm) than the PVA solution (128.43 ± 2.34 µS/cm), although the difference is smaller. However, when the gel-based ChCl:Urea DES-WK was blended with the PVA in different ratios, greatly increased conductivity values were observed, mainly to the PVA/ChCl:Urea DES-WK 70:30 (10,525.00 ± 229.81 µS/cm), Figure 3b. Thus, although the nanofiber diameters decrease with the increase in the conductivity of the electrospinning solution, by promoting the stretching of the jet, a too-high conductivity value will result in unstable jetting and consequently the formation of nanofibers will not occur [31].

#### 2.3.3. Viscosity

**Figure 4:**
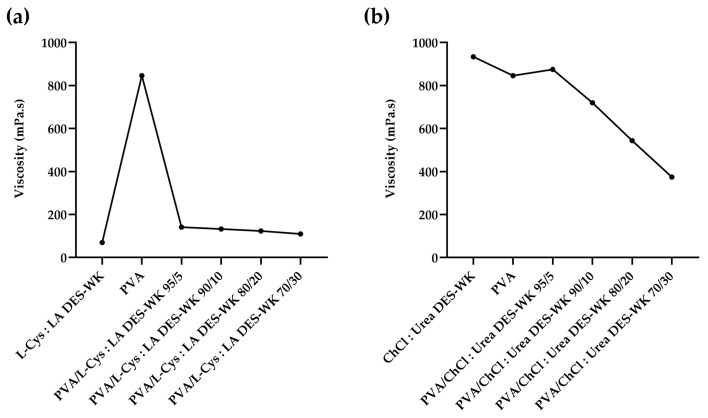
Viscosity measurements of the PVA/L-Cys:LA DES-WK blend gel solutions (**a**) and PVA/ChCl:Urea DES-WK blend gel solutions (**b**), as well as that of raw PVA and gels based on DES-WK mixtures.

The viscosity of the electrospinning solution, an important measure of the polymer chain entanglements, is another parameter that has a significant impact on the diameter and shape of the nanofibers produced through the electrospinning technique. In addition, it is affected by the temperature, and depends on the concentration of the solution and the molecular weight of the polymer, since higher values result in densely entangled polymer chains, and consequently in more viscous solutions. Moreover, highly viscous solutions can make more difficult the elongation of the jet, thus leading to thick nanofibers, while low-viscosity solutions can result in jets that break up easily into droplets [32,33]. Therefore, a proper chain entanglement should be established in order to keep the solution jet coherent during the electrospinning process. In this sense, to produce high-quality nanofibers, the viscosity of gels based on DES-WK mixtures, PVA solution, and gel-based PVA/DES-WK blends in different ratios was measured, and the results are shown in Figure 4.

As expected, the PVA solution exhibited a high viscosity value (846 mPa.s), which was quite similar to the viscosity found in another previous study of the PVA when used to produce electrospun fiber mats (810 mPa.s) [34]. Additionally, L-Cys:LA DES-WK gel solution revealed a low viscosity (70 mPa.s), and therefore, blends with different weight ratios of PVA increased the chain entanglements and resulted in improved viscoelastic force, which can be enough to prevent breakup of the electrically charged jets. In this sense, the viscosity obtained for the PVA/L-Cys:LA DES-WK gel blends slightly increased with increasing amounts of PVA, with the maximum being found for the 95:5 ratio (141.2 mPa.s), Figure 4a. On the other hand, the gel based on ChCl: Urea DES-WK solution presented the highest viscosity (934 mPa.s), and consequently, after mixing with the PVA solution at different ratios, the viscosity remained high, particularly for the 95:5 ratio (875 mPa.s), Figure 4b.

Therefore, the effect of the solution viscosity on the quality of the nanofibers, and their diameters was explored in Section 2.4.1.

### 2.4. Characterization of the Gel-Based Electrospun PVA/DES-WK Nanofibrous Membranes

#### 2.4.1. Characterization of the Gel-Based Nanofibers’ Surface Morphology through Scanning Electron Microscopy (SEM) Analysis

In recent years, electrospun nanofibers have become the main target of different studies for the development of nanofibrous materials for a wide a range of applications. In this study, an electrospinning technique (the needle-free Nanospider^TM^ technology) was used to produce gel-based nanofibers based on WK dissolved in DES mixtures as a novel and sustainable approach for wool waste valorization. For that purpose, the effect of the pH and the properties of the electrospinning solutions (e.g., electrical conductivity and viscosity) were evaluated. However, in addition to the solution properties (e.g., electrical conductivity, concentration, viscosity, and surface tension), there are other parameters that can influence the electrospinning process, and consequently the features of the produced nanofibers, namely the processing variables (e.g., applied voltage, the distance between the electrode and the collector, electrode type, and flow rate) and the environmental conditions (e.g., temperature and humidity). In this regard, the electrospinning was performed under controlled processing conditions, namely by applying a high voltage of 80 kV, a collecting distance of 13 cm, and an electrode rotation rate of 55 Hz (electrode spin = 8.8 r/min).

In fact, both gels based on DES-WK showed poor electrospinnability, since L-Cys:LA DES-WK exhibited a low viscosity (70 mPa.s) and electrical conductivity (62.58 ± 11.97 mS/cm), which can lead to the formation of the unstable jets. In turn, the ChCl:Urea DES-WK revealed the highest viscosity (934 mPa.s) and a conductivity of 90.71 ± 20.48 mS/cm; however, it was not enough to also produce a stable electrospinning jet. Moreover, the wool, which is rich in many different functional groups, can establish diverse inter- and intramolecular bonds (e.g., ionic interactions, hydrogen bonds, van der Waals forces), and consequently, several complications may arise in forming the Taylor cone and drawing the nanofibers, given that a higher voltage is required to stretch the solution jet [35]. In this sense, the addition of the PVA circumvented the difficulty of directly electrospinning the gel-based DES-WK solutions, due to their suitability for application as a base polymer for designing electrospun nanofibrous structures. Predominantly, PVA has been extensively investigated due to the use of water-based solvents, along with their good processability, biocompatibility, biodegradability, chemo-thermal stability, mechanical performance, and low cost [36].

**Figure 5:**
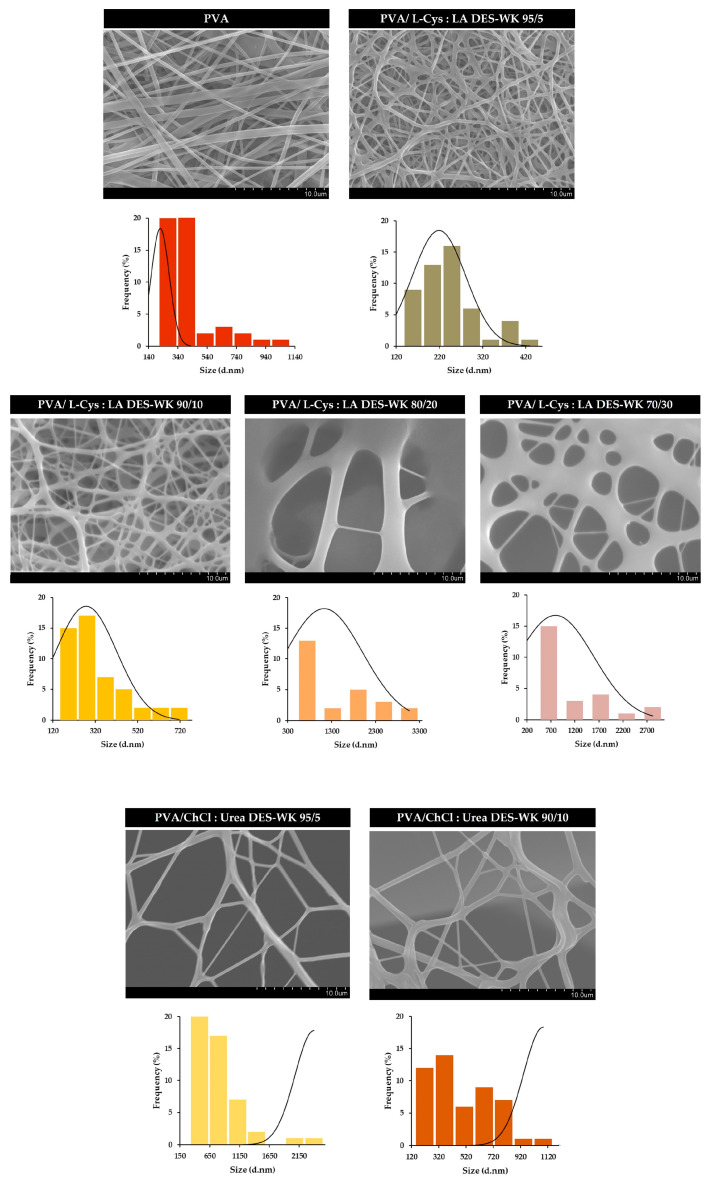
SEM images and fiber diameter distribution of the produced gel-based electrospun PVA/DES-WK nanofibrous membranes.

Hence, the formation and morphology of the electrospun nanofibers were analyzed through SEM, and the fiber diameters were determined using the ImageJ software, Figure 5.

The PVA showed a highly interconnected structure composed of fibers with a mean diameter of 346.68 ± 123.73 nm. In addition, when the PVA was added to the gel-based DES-WK solutions, the ability to form high-quality nanofibers was improved; in particular, the electrospinning solution composed of PVA/L-Cys:LA DES-WK 95/5 resulted in uniform fibers with a mean diameter of 219.14 ± 61.83 nm. Additionally, when the L-Cys:LA DES-WK ratio in the gel blends was increased to 80/20 and 70/30, this resulted in the formation of nanofibers without a smooth surface and with a wide distribution of fiber diameters due to the increase in the conductivity of the electrospinning solutions and slight decrease in the viscosity when increasing the content of L-Cys:LA DES-WK in the blends with the PVA (please see Section 2.3). On the other hand, the gel based on ChCl:Urea DES-WK was unable to produce nanofibers when the ratio of ChCl:Urea DES-WK increased to 80/20 and 70/30. However, the PVA/ChCl:Urea DES-WK 95/5 and the PVA/ChCl:Urea DES-WK 90/10 resulted in low fiber deposition. Therefore, under these conditions, the electrospinning solutions presented extremely high conductivity values, which prevented the formation of the stable jets, mainly under non-spinnable conditions (80/20 and 70/30).

Overall, the SEM images revealed that L-Cys:LA DES-WK was a more promising alternative for producing gel-based WK nanofibers from the wool waste.

Similarly, previous studies obtained WK nanofibers from the blends with polymers with a good fiber-forming capability, such as PVA, Polyethylene oxide (PEO), and Polycaprolactone (PCL). Nonetheless, the extraction of keratin from the wool has mainly been performed through sulfitolysis and by using toxic and harmful chemical reagents [15,16,18]. Thus, this is a more sustainable approach, which requires the use of DES as a kind of green solvent for WK dissolution. In addition, the present study directly uses wool dissolved in the DES mixtures in the electrospinning, without performing the WK extraction step, which makes the process more efficient, fast, and cost effective. Hence, this approach opens numerous possibilities for the development of gel-based electrospun nanofibrous membranes with potential in many biomedical and other industrial applications, and contributes to a sustainable future, in alignment with UN sustainability goals.

#### 2.4.2. Fourier-Transform Infrared Spectroscopic (FTIR) Analysis

**Figure 6:**
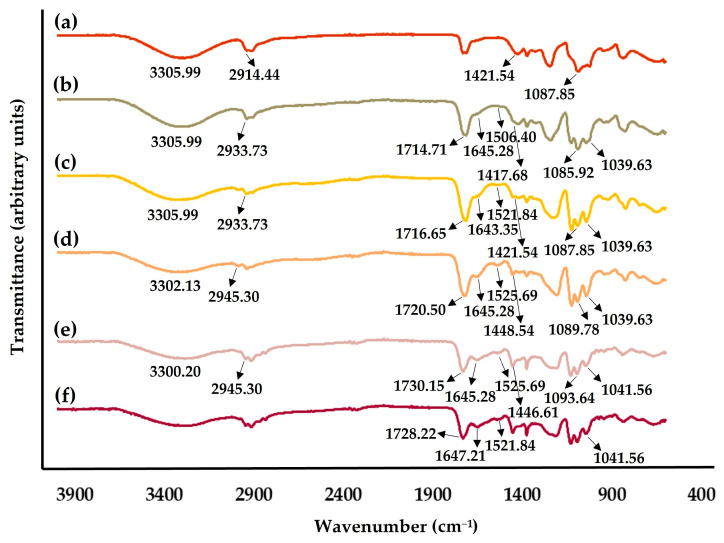
FTIR of the produced electrospun nanofibrous membranes and their raw materials: PVA (**a**), PVA/L-Cys:LA DES-WK 95/5 (**b**), PVA/L-Cys:LA DES-WK 90/10 (**c**), PVA/L-Cys:LA DES-WK 80/20 (**d**), PVA/L-Cys:LA DES-WK 70/30 (**e**), and L-Cys:LA DES-WK (**f**).

The chemical composition of the gel-based electrospun nanofibers produced using the blends of PVA and the gel based on L-Cys:LA DES-WK, which arose as a highly promising choice, was examined by FTIR analysis. In this regard, the acquired FTIR spectra of the raw PVA and the gel based on L-Cys:LA DES-WK, as well as the produced PVA/L-Cys:LA DES-WK gel blends, are presented in Figure 6.

The spectrum of the PVA exhibits characteristic peaks at 3305.99 cm^−^^1^ (-OH stretching vibration), 2914.44 cm^−^^1^ (CH stretching vibration), 1421.54 cm^−^^1^ (CH_2_ bending vibration), and 1087.85 cm^−^^1^ (C-O stretching vibration); Figure 6a [37]. In addition, the gel based on L-Cys:LA DES-WK shows the typical bands of the wool waste at 1647.21 cm^−^^1^ (amide I), 1521.84 cm^−^^1^ (amide II), and 1041.56 cm^−^^1^ due to the presence of cysteine-S-sulfonated residues [2], as well as a characteristic peak of the LA at 1728.22 cm^−^^1^ (C=O stretching vibration), one of the main constituents of the DES mixture, Figure 6f [38].

Similarly the spectra of the PVA/L-Cys:LA DES-WK gel blends display the typical peaks of the PVA at around 3300 cm^−^^1^, 2930 cm^−^^1^, 1421 cm^−^^1^, and 1087 cm^−^^1^, as well as the characteristic bands of the gel based on L-Cys:LA DES-WK at around 1720 cm^−^^1^, 1645 cm^−^^1^, 1520 cm^−^^1^, and 1041 cm^−^^1^, which indicates that the protein functional groups/structure of the wool was preserved even after WK dissolution in the L-Cys:LA DES mixture. In addition, the peaks of wool waste at around 1640 cm^−^^1^ and 1518 cm^−^^1^ are more evident when increasing of L-Cys:LA DES-WK ratio, while the intensity of the broad absorption peak at 3319 cm^−^^1^ that belong to PVA decrease, Figure 6.

Furthermore, after cross-checking the acquired FTIR spectra with the LabSolutionsIR library (Table 2), it was observed that the PVA/L-Cys:LA DES-WK 95/5 and the PVA/L-Cys:LA DES-WK 90/10 were matched with the PVA, while the PVA/L-Cys:LA DES-WK 80/20, the PVA/L-Cys:LA DES-WK 70/30, and the gel based on L-Cys:LA DES-WK were matched with the ethyl lactate, probably due to the higher L-Cys:LA ratio in these nanofibers. Thus, it can be concluded that some characteristic peaks of the PVA/L-Cys:LA DES-WK nanofibers can overlap with bands of L-Cys:LA DES mixture and PVA.

#### 2.4.3. Characterization of the Gel-Based Nanofibers’ Mechanical Properties

In this study, the mechanical properties, namely the Young’s modulus, tensile strength, and elongation at break, were evaluated in dry conditions for the raw PVA and the gel-based electrospun PVA/L-Cys:LA DES-WK nanofibrous membranes. The values are presented Table 3.

PVA, a synthetic polymer well known by its mechanical strength, showed a higher Young’s modulus (45.04 ± 3.58 MPa) and tensile strength (8.18 ± 1.25 MPa) than the PVA/L-Cys:LA DES-WK gel blends, which had a weaker mechanical performance. However, the tensile strength and Young’s modulus of the PVA/L-Cys:LA DES-WK gel blends were not significantly affected when the ratio of gel based on DES-WK increased from 95/5 to 90/10, resulting in tensile strengths of 4.19 ± 0.96 MPa and 4.43 ± 1.14 MPa and Young’s Modulus of 22.24 ± 3.00 MPa and 27.01 ± 0.18 MPa, respectively. On the other hand, the elongation at break assays revealed that the PVA, the PVA/L-Cys:LA DES-Wool 95/5, and the PVA/L-Cys:LA DES-Wool 90/10 could bear strains of 18.34 ± 3.97%, 19.28 ± 8.05%, and 16.40 ± 5.99, respectively. Thus, the produced gel-based electrospun nanofibrous membranes presented similar elongation to break values, although a slight increase (not significant) was noted when increasing the PVA content from 90/10 to 95/5.

Therefore, the mechanical properties exhibited by the gel-based electrospun PVA/L-Cys:LA DES-WK nanofibrous membranes emphasized their suitability for use in biomedical and other industrial applications.

#### 2.4.4. Evaluation of the Gel-Based Nanofibers’ Antioxidant Activity

The antioxidant activity of the produced gel-based electrospun PVA/L-Cys:LA DES-WK nanofibrous membranes was explored through ABTS assay. ABTS+ is a stable free radical commonly used for assessing the total antioxidant capacity of natural compounds, and displays a maximum absorption peak at 734 nm [39,40].

**Figure 7:**
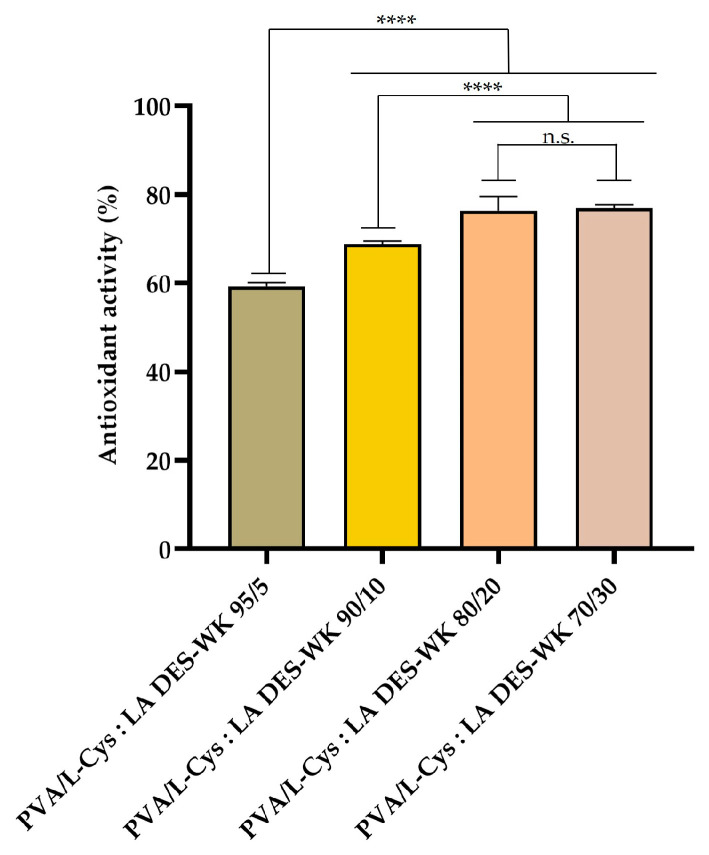
Schematic representation of the antioxidant activity of the produced gel-based electrospun PVA/L-Cys:LA DES-WK nanofibrous membranes. (Data are presented as the mean ± standard deviation, n.s. *p* > 0.05, **** *p* < 0.0001).

The results presented in Figure 7 show that the antioxidant activity improved with increasing L-Cys:LA DES-WK ratio. The PVA/L-Cys:LA DES-WK 95/5 exhibited a value of 59.25 ± 0.01%, while the PVA/L-Cys:LA DES-Wool 70/30 exhibited a value of 77.07 ± 0.01%. The improvement in the antioxidant activity of the gel-based electrospun nanofibrous membranes is attributed to the components of the DES mixture, namely to the L-Cys and LA, as well as the WK. In fact, in the literature, Nogueira et al. highlighted the antioxidant activity of the L-Cys [25], while Hu et al. revealed that LA-producing bacteria have prominent antioxidant properties [41]. Moreover, the WK displays a high cysteine content, antioxidant functions of which have been demonstrated previously [26]. Therefore, the promising antioxidant capability of the produced gel-based electrospun PVA/L-Cys:LA DES-WK nanofibrous membranes arises from the ability of the DES mixture and the WK to donate a hydrogen atom to free radicals, reducing the ABTS+ radical to a colorless compound [40].

#### 2.4.5. Evaluation of the Gel-Based Nanofibers’ Antimicrobial Properties

In this study, the antimicrobial properties of the produced gel-based electrospun PVA/L-Cys:LA DES-WK nanofibrous membranes were characterized by using *S. aureus* and *K. pneumoniae* as Gram-positive and Gram-negative bacteria models, respectively.

**Figure 8:**
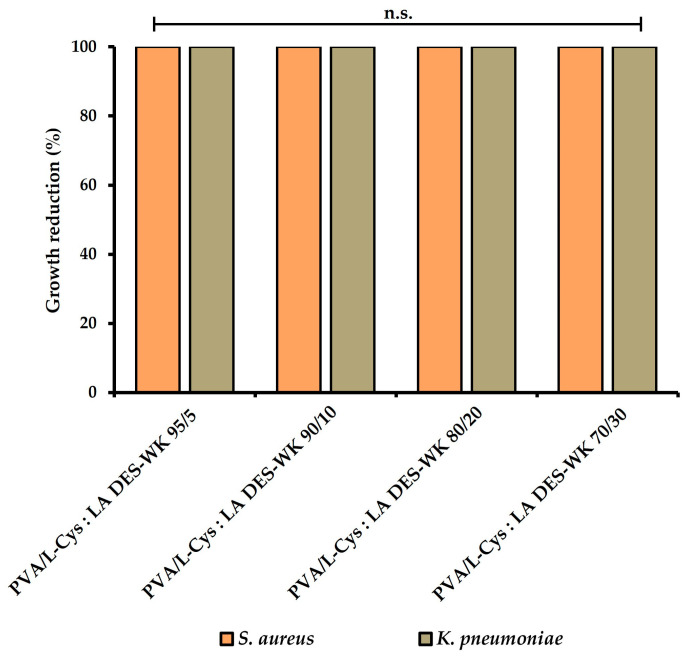
Schematic representation of the antibacterial activity of the produced gel-based electrospun PVA/L-Cys:LA DES-WK nanofibrous membranes (n.s. *p* > 0.05).

The results presented in Figure 8 and Table 4 show that the PVA/L-Cys:LA DES-WK gel blends exhibited an inhibitory effect of 100% (reaching a 6 Log reduction) on *S.aureus* and *K. pneumoniae* growth, indicating a significant statistical difference with the control group, where the raw PVA was used. Therefore, the synergistic effect of the intrinsic antimicrobial activity of the L-Cys:LA DES and WK resulted in the production of gel-based nanofibers with exceptional antibacterial properties. In addition, these results support the antibacterial activity of the gels based on DES-WK obtained in Section 2.2, revealing inhibition of *S. aureus* and *K. pneumoniae* growth due to the antimicrobial properties of L-Cys:LA DES and WK.

In the literature, it has already been described that the sulfhydryl functional groups (-SH) of the L-Cys interact with the -SH groups present at the cell membrane proteins, leading to a great decrease in enzymatic activity and bacterial metabolism [25]. Likewise, LA is lethal to microorganisms when undissociated molecules enter the cell through the cell membrane and ionize inside. Moreover, the acidic pH compromises enzymatic and protein activities, as well as inducing DNA damage, disrupting the integrity of the bacterial membrane. Additionally, the LA can cause changes in the cell membrane structure and permeability, leading to the leakage of the cellular content [26]. Additionally, the WK is known for displaying a high cysteine content, and thus the wool waste also contributes to the antibacterial activity presented by the produced gel-based nanofibrous membranes [26].

Furthermore, it is important to emphasize that the percentages of bacterial reduction obtained are in accordance with the indications of the US FDA and their European counterparts, which consider that a bacterial reduction of at least 99.99% should be achieved for it to be considered as having antimicrobial properties [40]. Moreover, Wang et al. extracted WK from wool waste following the reduction-C method, which uses organic solvents, and blended it with PCL to produce nanofibers through electrospinning. The data obtained in this study showed a similar antibacterial effect against *S. aureus* and *Escherichia coli* (*E. coli*) (100% bacterial reduction) only when the electrospun PCL/WK nanofibers were chlorinated in diluted sodium hypochlorite solution, emphasizing the suitability of the produced PVA/L-Cys: LA DES-WK nanofibers for application as an antimicrobial material in various fields [18].

## 3. Conclusions

Despite the tremendous efforts made in the textile industry, the generation of textile waste remains a serious problem. To overcome this situation, several studies have been performed focusing on recycling and remanufacturing this waste in order to produce new materials. Herein, a green and sustainable approach was used to dissolve the WK from textiles with DES, a new class of natural, non-toxic, eco-friendly, and biodegradable solvents that have the unique ability to dissolve and regenerate WK. In addition, after confirming the potential of both ChCl:Urea and L-Cys:LA DES mixtures in the dissolution of the WK, the gels based on DES-WK were for the first time directly prepared by electrospinning, without further extraction steps. For that purpose, and to overcome the challenge of electrospinning the WK biopolymer, PVA, a water-soluble and biodegradable polymer widely used for nanofiber production, was blended with the gels based on DES-WK in different ratios. The pH and the properties of the electrospinning solutions, such as electrical conductivity and viscosity, were measured in order to evaluate their influence on the morphology and properties of the electrospun nanofibers. The results revealed that the L-Cys:LA DES containing dissolved WK was more suitable for electrospinning with the PVA, with uniform fibers being observed, particularly for the PVA/L-Cys:LA DES-WK 95/5. Additionally, the FTIR spectra of the produced nanofibrous membranes presented the characteristic peaks of both PVA and the gel based on L-Cys:LA DES-WK, which indicates that the WK was preserved even after dissolution in the L-Cys:LA DES mixture. Moreover, the gel-based electrospun PVA/L-Cys:LA DES-WK nanofibrous membranes displayed remarkable antioxidant and antimicrobial properties. Hence, in this study, we intended to find solutions promoting textile sustainability and to propose a cascading approach valorization strategy for dissolving WK and fabricating WK gel-based nanofibers through electrospinning with potential in many biomedical and other industrial applications, such as in cell-growth scaffolds, tissue engineering and regeneration, intelligent electronic components, and in agricultural applications.

## 4. Materials and Methods

### 4.1. Materials

Wool waste was obtained from “A Transformadora, Lda”, a wool finishing manufacturer in Covilhã, Portugal. L-Cysteine (L-Cys), Urea, Mueller–Hinton Broth (MHB), Nutrient Agar (NA), Nutrient Broth (NB), Resazurin (7-hydroxy-3H-phenoxazin-3-one 10-oxide) dye, Tween 80, and Sodium chloride (NaCl) were purchased from Sigma-Aldrich (Sigma-Aldrich, St. Louis, MO, USA). DL-Lactic acid 90% (LA), Choline chloride 99% (ChCl) were acquired from Fisher Scientific (Fisher Scientific, Leicestershire, UK). Poly(vinyl alcohol) PVA (115,000 g/mol) was provided from VWR Chemicals (VWR Chemicals, Leuven, Belgium). ABTS was purchased from Panreac (Panreac, Barcelona, Spain). Potassium persulfate was acquired from Acros Organics (Acros Organics, Geel, Belgium).

### 4.2. Preparation of DES Mixtures

The L-Cys:LA DES was prepared as previously described by Okoro et al. [2]. Briefly, 1.6 g of L-Cys and 20 mL of LA were stirred at 105 °C into a lab flask until a homogeneous DES mixture was obtained. In turn, the ChCl:Urea DES was produced by mixing the two components at a 1:2 molar ratio, under stirring at 80 °C, until a transparent and homogenous liquid was obtained, as described by Jiang et al. [3].

### 4.3. Dissolution of WK into DES Mixtures

The WK dissolution was carried out through the immersion of 2.0 g of wool waste in the previously prepared L-Cys:LA DES. In turn, 0.16 g of wool waste was immersed into ChCl:Urea DES by adapting a protocol previously reported by Jiang et al. [3]. Both DES mixtures were stirred at 130 °C for 3 h, until dissolution was complete and homogenous gel solutions were obtained. The solubility of the gels based on DES-WK was determined through Equation (1):

(1) $$Solubility\left(\%\right)=\frac{W_{0}-W_{1}}{W_{0}}\times 100$$

where *W*_0_ is the weight of wool waste before dissolution and *W*_1_ is the weight of wool waste after dissolution.

### 4.4. Determination of the Antibacterial Activity of the DES Mixtures and the Gels Based on DES-WK

The antibacterial activities of the DES mixtures and the gels based on DES-WK solutions were characterized by broth microdilution assay according to CLSI M07-A6 guidelines using *Staphylococcus aureus* (*S. aureus*) (ATTC 6538) and *Klebsiella pneumoniae* (*K. pneumoniae*) (ATCC 4352) as model bacterial. Briefly, sequential dilutions of the DES mixtures and the DES-WK gel solutions (e.g., ChCl:Urea DES, L-Cys:LA DES, L-Cys:LA DES-WK, and ChCl:Urea DES-WK) were prepared in sterile MHB. Then, overnight liquid suspensions of *S. aureus* and *K. pneumoniae* were adjusted in sterile water to 1 × 10^8^ CFU/mL (0.5 McFarland turbidity) and further diluted 1:10 in MHB to prepare bacteria work suspensions of 1 × 10^7^ CFU/mL. After that, a volume (50 µL) of the work suspension of the *S. aureus* and *K. pneumoniae* and 50 µL of the serial dilutions of DES and the DES-WK gel solutions were pipetted into 96-well plates and incubated for 24 h at 37 °C. After incubation, 30 µL of the 0.02% (*w/v*) resazurin solution were added to the 96-well plates and further incubated for 4 h. The lowest concentration of the prepared dilutions that inhibit the bacteria growth was defined by a resazurin color change from blue to pink. In this sense, a reduction by viable cells of the resazurin blue dye in its oxidized state to a pink, fluorescent resorufin product indicated bacterial growth. Wells containing only MHB medium were included as a negative control (K−), whereas wells filled with MHB medium and bacterial work suspensions were used as positive control (K+).

### 4.5. Production of the Gel-Based Electrospun PVA/DES-WK Nanofibrous Membranes

#### 4.5.1. Preparation of the Electrospinning Solutions

For the electrospinning solutions, 10% PVA (*w/v*) with a molecular weight of 115,000 g/mol was dissolved in distilled water at 90 °C and kept under magnetic stirring overnight until complete dissolution. After that, the PVA was added to DES-WK gel solutions with PVA/DES-WK blending ratios of 95/5, 90/10, 80/20, and 70/30. The blends were stirred continuously for an additional 2 h to obtain homogeneous PVA/DES-WK blend gel solutions.

#### 4.5.2. Measurement of pH of the Electrospinning Gel Solutions

The pH of the PVA, the gels based on DES-WK, and the PVA/DES-WK blend gel solutions was monitored throughout using a digital pH meter (Mettler Toledo Seven Easy pH Meter). The pH value was read after reaching a constant state. All determinations were performed in triplicate.

#### 4.5.3. Measurement of the Electrical Conductivity of the Electrospinning Gel Solutions

The electrical conductivity of the PVA, the gels based on DES-WK, and each PVA/DES-WK blend gel solution was measured at room temperature using a digital conductivity meter (Mettler Toledo FiveEasy Conductivity Meter, Columbus, OH, USA). The conductivity values were read from the digital screen of the conductivity meter when it stabilized. The measurements were carried out in triplicate.

#### 4.5.4. Measurement of the Viscosity of the Electrospinning Gel Solutions

The viscosity of the PVA, the gels based on DES-WK, and the PVA/DES-WK blend gel solutions were determined with a rotational viscometer (VR 3000 MYR, model V1-L, Viscotech Hispania SL., Tarragona, Spain). For each solution, several types of spindles (TL5, TL6, and TL7) were used with different rotation speeds to accurately measure the solutions’ viscosity. Both the temperature of the solutions and room temperature were checked before and after each measurement. All measurements were carried out in triplicate.

#### 4.5.5. Electrospinning of the PVA/DES-WK Blend Gel Solutions

The raw PVA and the PVA/DES-WK blend gel solutions were electrospun using a modified electrospinning technique, Nanospider Technology (Nanospider laboratory machine NS LAB 500S from Elmarco s.r.o., Liberec, Czech Republic, http://www.elmarco.com, accessed on 12 August 2023). Electrospinning was carried out with a working distance (distance from the spinning electrode to the collector) of 13 cm, an applied voltage of 80.0 kV, and an electrode rotation rate of 55 Hz. The collection time was ~30 min on polypropylene nonwoven fabric at 25 °C.

**Figure 9:**
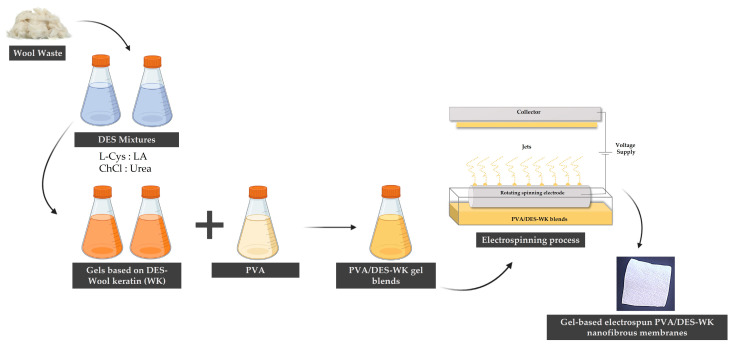
Schematic overview of the fabrication process of the gel-based electrospun PVA/DES-WK nanofibrous membranes.

The fabrication of the gel-based electrospun PVA/DES-WK nanofibrous membranes is overviewed in Figure 9.

### 4.6. Characterization of the Gel-Based Electrospun PVA/DES-WK Nanofibrous Membranes

#### 4.6.1. Characterization of the Gel-Based Nanofibers’ Surface Morphology through Scanning Electron Microscopy (SEM) Analysis

The surface morphology of the gel-based electrospun PVA/DES-WK nanofibrous membranes was characterized through SEM. Briefly, all samples were placed on aluminum stubs, fixed with Araldite adhesive, and sputter coated with a thin layer of gold using a Quorum Q150R ES sputter coater (Quorum Technologies Ltd., Laughton, East Sussex, UK) prior to SEM observation. After that, the SEM images were taken with a Hitachi S-3400N Scanning Electron Microscope (Hitachi, Tokyo, Japan) using an acceleration voltage of 20 kV. The morphology of the nanofibers was analyzed by SEM images, and the fibers’ diameters were measured using an image analysis software, ImageJ (NIH Image, Bethesda, MD, USA). The raw PVA was also analyzed for comparative purposes.

#### 4.6.2. Fourier-Transform Infrared Spectroscopic (FTIR) Analysis

Fourier-transform infrared spectra of the raw materials (PVA and the gel based on L-Cys:LA DES-WK) and the gel-based electrospun PVA/L-Cys:LA DES-WK nanofibrous membranes were acquired on an IRAffinity-1S FTIR spectrophotometer (Shimadzu, Kyoto, Japan). Data were collected with an average of 64 scans, in the range of 4000–600 cm^−1^, and a spectral resolution of 4 cm^−1^. All the samples were then analyzed and compared against the LabSolutionsIR (Version 2.11, Shimadzu, Kyoto, Japan) library.

#### 4.6.3. Characterization of the Gel-Based Nanofibers’ Mechanical Properties

The mechanical performance of the PVA and gel-based electrospun PVA/L-Cys:LA DES-WK nanofibrous membranes was analyzed with a universal tensile test machine (DY-35, Adamel Lhomargy, Roissy en Brie, France) operated at room temperature, under dry conditions, equipped with a 100 N static load cell. For the test, electrospun nanofiber samples were cut into strip-shaped specimens with a width of 1 cm and a gauge length of 4 cm, and their thickness, ranging from 0.174 to 0.292 mm, was measured with a micrometer (Adamel Lhomargy MI20, Draveil, France). The length between the clamps and the speed of testing were set to 1 cm and 1 mm/min, respectively. On each sample, measurements were repeated three times, and the average value was recorded.

#### 4.6.4. Evaluation of the Gel-Based Nanofibers’ Antioxidant Activity

The antioxidant activity of the gel-based electrospun PVA/L-Cys:LA DES-WK nanofibrous membranes was assessed using the ABTS radical decolorization assay. Briefly, the ABTS radical cation (ABTS+) was initially formed by mixing 5 mL of ABTS (7 mM) stock solution with 88 µL of potassium persulfate (2.4 mM). The reaction mixture was then incubated in the dark for 12–16 h at room temperature. Prior to the beginning of the assay, the ABTS+ solution was diluted with phosphate buffer (0.1 M, pH 7.4) to reach an absorbance of 0.700 ± 0.025, at 734 nm. Then, the reaction occurred for 30 min in the dark after emerging 10 mg of each gel-based electrospun nanofibers sample in 10 mL of ABTS+ solution. The scavenging capability of ABTS+ at 734 nm was determined through Equation (2):

(2) $$Antioxidant\;activity\;\left(\%\right)=\frac{A_{control}-A_{sample}}{A_{control}}\times 100$$

where *A_control_* is the absorbance of the remaining ABTS+ in the control sample (e.g., PVA) and *A_sample_* is the absorbance of the remaining ABTS+ when incubated with the nanofiber’s samples (e.g., gel-based electrospun PVA/L-Cys:LA DES-WK nanofibrous membranes). All experiments were conducted in triplicate.

#### 4.6.5. Evaluation of the Gel-Based Nanofibers’ Antimicrobial Properties

The ability of the produced gel-based electrospun PVA/L-Cys:LA DES-WK nanofibrous membranes to inhibit bacteria growth was characterized using *S. aureus* (ATCC 6538) and *K. pneumoniae* (ATCC 4352) on the basis of Japanese Industrial Standard JIS L 1902:2002. For this purpose, bacterial suspensions (1 × 10^5^ CFU/mL) were prepared by transferring fresh colonies from overnight cultures into sterile NB and inoculating them over the nanofibers’ samples. For this purpose, the samples were evaluated immediately after adding the inoculum (T0h) and after 18–24 h in contact with the inoculum at 37 °C (T24h). Then, each sample was subjected to vigorous vortexing for 30 s in a neutralizing solution composed of 0.85 (*w/v*) NaCl and 2 mL/L of Tween 80, and serial dilutions were prepared with 0.85 (*w/v*) NaCl and seeded on NA plates. The bacterial colonies were counted after overnight incubation at 37 °C and expressed as CFU/mL. The percentage of bacterial growth inhibition was determined accordingly with Equation (3):

(3) $$Antibacterial\;activity\left(\%\right)=\frac{C-S}{C}\times 100$$

where *C* and *S* represent the CFU/mL of control (e.g., PVA) and experimental group (e.g., gel-based electrospun PVA/L-Cys:LA DES-WK nanofibrous membranes), respectively.

#### 4.6.6. Statistical Analysis

The statistical data analysis was conducted using GraphPad Prism 6 software (GraphPad Software, La Jolla, CA, USA) through a one-way analysis of variance (ANOVA), followed by Tukey’s multiple comparison test. A *p* value lower than 0.05 (*p* < 0.05) was considered statistically significant. All experiments were conducted in triplicate unless otherwise stated, and the data expressed as a mean ± standard deviation (SD).

## Figures and Tables

**Table 1:** Solubility of wool keratin (WK) in the DES mixtures.

DES Mixture	Time (h)	T (°C)	Solubility (%)
ChCl:Urea molar ratio 1:2	3	130	42.88 ± 0.83
1.6 g L-Cys in 20 mL LA	3	130	68.83 ± 5.10

**Table 2:** Matches found using the LabSolutionsIR library for all nanofibers produced.

Sample	Degree of Confidence	Corresponding Polymer/Solvent
PVA	855—Medium	PVA
PVA/L-Cys LA DES-WK 95/5	825—Medium	PVA
PVA/L-Cys LA DES-WK 90/10	786—Medium	Ethyl Lactate
PVA/L-Cys LA DES-WK 80/20	805—Medium	Ethyl Lactate
PVA/L-Cys LA DES-WK 70/30	752—Medium	Ethyl Lactate
L-Cys LA DES-WK	758—Medium	Ethyl Lactate

**Table 3:** Characterization of the mechanical properties of the produced gel-based electrospun PVA/L-Cys:LA DES-WK nanofibrous membranes and associated PVA raw material.

	Tensile Strength (MPa)	Young’s Modulus (MPa)	Elongation at Break (%)	Thickness (mm)
PVA	8.18 ± 1.25	45.04 ± 3.58	18.34 ± 3.97	0.174 ± 0.02
PVA/ L-Cys:LA DES-WK 95/5	4.19 ± 0.96	22.24 ± 3.00	19.28 ± 8.05	0.292 ± 0.01
PVA/ L-Cys:LA DES-WK 90/10	4.43 ± 1.14	27.01 ± 0.18	16.40 ± 5.99	0.210 ± 0.02
PVA/ L-Cys:LA DES-WK 80/20 *	-	-	-	-
PVA/ L-Cys:LA DES-WK 70/30 *	-	-	-	-

* It was not possible to remove the nanofibers from the collector due to their poor quality.

**Table 4:** Antibacterial efficiency of the electrospun gel-based PVA/L-Cys:LA DES-WK nanofibrous membranes against *S. aureus* and K. pneumoniae expressed as percentage of bacterial reduction (%R).

		*S. aureus*			*K. pneumoniae*	
Samples		CFU/mL	Growth Reduction (%)		CFU/mL	Growth Reduction (%R)
PVA	0 h	7.25 × 10^3^	-	0 h	3.63 × 10^4^	-
	24 h	3.02 × 10^8^	-	24 h	2.94 × 10^8^	-
PVA/L-Cys:LA DES-WK 95/5		0.00 × 10^0^	100.00%		0.00 × 10^0^	100.00%
PVA/L-Cys:LA DES-WK 90/10		0.00 × 10^0^	100.00%		0.00 × 10^0^	100.00%
PVA/L-Cys:LA DES-WK 80/20		0.00 × 10^0^	100.00%		0.00 × 10^0^	100.00%
PVA/L-Cys:LA DES-WK 70/30		0.00 × 10^0^	100.00%		0.00 × 10^0^	100.00%

## Data Availability

Data that support the findings of this study are included in the article.
